# Occurrence and Genetic Characteristics of *Cryptosporidium hominis* and *Cryptosporidium andersoni* in Horses from Southwestern China

**DOI:** 10.1111/jeu.12399

**Published:** 2017-03-08

**Authors:** Lei Deng, Wei Li, Zhijun Zhong, Chao Gong, Xuefeng Cao, Yuan Song, Wuyou Wang, Xiangming Huang, Xuehan Liu, Yanchun Hu, Hualin Fu, Min He, Ya Wang, Yue Zhang, Kongju Wu, Guangneng Peng

**Affiliations:** ^1^ The Key Laboratory of Animal Disease and Human Health of Sichuan Province, College of Veterinary Medicine Sichuan Agricultural University Chengdu Sichuan Province 611130 China; ^2^ Chengdu Giant Panda Breeding Research Base Chengdu Sichuan Province 625001 China

**Keywords:** *GP60*, multilocus sequence typing, transmission

## Abstract

A total of 333 fecal specimens from horses in southwestern China were genotyped based on analysis of the small subunit rRNA (*SSU* rRNA) gene. *Cryptosporidium hominis* and *Cryptosporidium andersoni* were identified in 2 and 4 stool specimens, respectively. The identification of *C. hominis* was confirmed by sequence analysis of the 70‐kDa heat shock protein (*HSP70*) and oocyst wall protein (*COWP*) genes. Subtyping analysis of the 60‐kDa glycoprotein (*GP60*) gene sequence of *C. hominis* revealed a new rare subtype Id, named IdA15; only three Id isolates have been reported in humans to date. Multilocus sequence typing (MLST) analysis indicated that the *C. andersoni* subtype was A6, A5, A2, and A1 at the four minisatellite loci (MS1, MS2, MS3, and MS16, respectively). This is the first report to identify the presence of *C*. *andersoni* and *C*. *hominis* in horses in southwestern China and the first to identify a rare zoonotic subtype Id of *C*. *hominis* in horses. These findings suggest that infected horses may act as potential reservoirs of *Cryptosporidium* to transmit infections to humans.

*CRYPTOSPORIDIUM* spp. are common protozoan parasites that cause diarrhea in a wide variety of vertebrate animals. Although *Cryptosporidium* was initially discovered by Tyzzer in 1907, it was not recognized as an important parasite for the next 50 yr (Tyzzer 1907). Subsequently, *Cryptosporidium* was also identified in turkeys, calves and humans with diarrhea, and received more attention around the world (Current et al. 1983; Panciera et al. 1970; Slavin 1955). *Cryptosporidium* attracted great public health attention in the large waterborne outbreak in Milwaukee (U.S.A.) in 1993, where over 400,000 people were infected (Kenzie et al. 1995). In young calves, high morbidity caused by *Cryptosporidium* can lead to substantial production losses to the livestock industry (Graaf et al. 1999).

Most animals become infected through direct contact with an infected host, or through indirect ingestion of oocyst‐contaminated water or foods (Cacciò 2005; Fayer et al. 2000). Although clinical presentations of cryptosporidiosis are often self‐limiting or sometimes asymptomatic in healthy individuals, fatal and chronic diarrhea can occur in immunocompromised or immunosuppressed individuals (Petri et al. 2008). Recent molecular epidemiologic surveys of cryptosporidiosis from different host species have identified at least 30 valid species and more than 70 genotypes, with new genotypes continually being found (Ryan et al. 2014, 2015). Of these, *Cryptosporidium hominis* is primarily a human pathogen (Xiao et al. 2001), and *Cryptosporidium andersoni* is the major species responsible for bovine cryptosporidiosis (Liu et al. 2015).

In China, horses are commonly used for work, entertainment, and competitions, usually living in close association with humans. Cryptosporidiosis was initially described in immune‐deficient Arabian foals by Snyder et al. (1978). To date, nine *Cryptosporidium* species/genotypes have been identified in equines in more than eight countries (Liu et al. 2015). However, only limited reports about horse cryptosporidiosis are available in China, and the zoonotic transmission of *Cryptosporidium* between humans and horses remains unknown. Thus, the present study was conducted to identify *Cryptosporidium* species/genotypes in horses from southwestern China, and to assess the zoonotic potential of cryptosporidiosis from horses to humans.

## Materials and Methods

### Ethics statement

The present study protocol was reviewed and approved by the Research Ethics Committee and the Animal Ethical Committee of Sichuan Agricultural University. Permission was obtained from farm managers before the collection of fecal specimens from horses.

### Specimen collection

In total, 333 fecal samples from horses of different ages (3 mo to 16 yr) were randomly collected on five farms with different management systems in the Sichuan and Yunnan provinces of southwestern China between August 2015 and April 2016. In total, 100 horses from Farm 1 (48 horses) and Farm 2 (52 horses), which are both equestrian clubs, were kept in stables; 56 horses from Farm 3 were kept in pastures; 12 horses from Farm 4, mainly used for experimental research, were kept in stables; and 165 horses from Farm 5 were used for breeding in pastures and stables. Each sample was collected using a sterile disposable latex glove immediately after the horse defecated onto the ground, and was then placed into an individual sterile plastic container. No obvious clinical signs were observed in any of the sampled horses. Samples were maintained at 4 °C until DNA extraction.

### DNA extraction

Approximately 0.5 g of each of the stored fecal specimens was washed three times with distilled water by centrifugation at 2,000 *g* for 10 min. DNA was extracted from the washed fecal samples, using an EZNA^®^ Stool DNA kit (Omega Biotek, Norcross, GA) according to the manufacturer's recommended protocol. DNA was eluted in 200 μl of the Solution Buffer of the kit and stored at −20 °C until used for polymerase chain reaction (PCR) analysis.

### *Cryptosporidium* genotyping and subtyping

*Cryptosporidium* species/genotypes were identified in all of the extracted DNA samples by nested PCR amplification of an ∼830‐bp fragment of the small subunit rRNA (*SSU* rRNA) gene (Xiao et al. 1999) (Table S1). The identification of *C. hominis* was confirmed by PCR and sequence analysis of the approximately 1,950‐ and 550‐bp fragments for the *Cryptosporidium* oocyst wall protein (*COWP*) and 70‐kDa heat shock protein (*HSP70*) genes, respectively (Sulaiman et al. 2000; Xiao et al. 2000) (Table S1). Subtype identification of *C. hominis* isolates was performed by sequence analysis of an approximately 800‐bp fragment of the 60‐kDa glycoprotein (*GP60*) gene, and *C. andersoni* isolates were determined by amplification at four loci MS1, MS2, MS3, MS16 (fragment lengths of approximately 550, 450, 530, and 590 bp, respectively). Primers and amplification conditions were adopted as previously described (Sulaiman et al. 2005; Feng et al. 2011) (Table S1).

### Nucleotide sequencing and analysis

All secondary PCR products were directly sequenced at Life Technologies (Guangzhou, China) using an ABI Big Dye Terminator v3.1 cycle sequencing kit (Applied Biosystems, Carlsbad, CA). The accuracy of the sequences was confirmed by bidirectional sequencing, and a new PCR secondary product was re‐sequenced if necessary. The nucleotide sequences obtained in this study were aligned with the corresponding *Cryptosporidium* reference sequences downloaded from the GenBank database, using BLAST (http://blast.ncbi.nlm.nih.gov) and ClustalX 1.83 (http://www.clustal.org/) to determine *Cryptosporidium* species/genotypes and subtypes.

### Phylogenetic analysis

Phylogenetic analysis was performed by constructing neighboring‐joining trees of the *GP60* genes, using the program Mega 6 (http://www.megasoftware.net/) based on the evolutionary distances calculated by the Kimura‐2‐parameter model. The reliability of the trees was assessed by bootstrap analysis with 1,000 replicates.

### Nucleotide sequence accession numbers

The unique partial nucleotide sequences of the *SSU* rRNA, *HSP70, COWP, GP60* genes and the four minisatellite loci (MS1, MS2, MS3, and MS16) obtained in the present study were deposited in the GenBank database under accession numbers KX926452–KX926463 and KY210530–KY210545, respectively.

## Results and Discussion

In the present study, six of the 333 (1.8%) fecal specimens were detected to be positive for *Cryptosporidium* by PCR amplification of the partial *SSU* rRNA gene, which is consistent with the previously reported low infection rate of *Cryptosporidium* in horses in Algeria (2.3%), Poland (3.4%), and New York state (5.1%) (Burton et al. 2010; Laatamna et al. 2015; Wagnerová et al. 2015). The age of the *Cryptosporidium*‐positive horses was between 2 and 3 yr. Analysis of the sequences of the *SSU* rRNA gene of the six *Cryptosporidium*‐positive samples revealed the presence of two *C. hominis* and four *C. andersoni* isolates. Comparison of the *SSU* rRNA gene sequences revealed that the two *C. hominis* isolates were identical to the *C. hominis* (KF679723) isolate derived from a rhesus macaque in China (Karim et al. 2014), and the three *C. andersoni* isolates were identical to the *C. andersoni* (KT175425) isolate derived from wastewater in Iran. However, the remaining sequence of *C. andersoni* had one nucleotide substitution (T/C at nucleotide 595) when compared to the *C. andersoni* (KF271453) isolate derived from a human in China. The *HSP70* sequence of one isolate had two nucleotide substitutions (T/G and A/T at positions 41 and 879, respectively) compared to the sequence (KF679727) derived from a rhesus macaque in China, but the other isolate was identical to the published *C. hominis* sequence KM116517 derived from a human in China. Interestingly, the two *C. hominis COWP* gene sequences obtained in this study were identical to each other, and were also identical to *C. hominis* isolates obtained from a human (DQ388389) and a monkey (AF266272).

**Figure 1:**
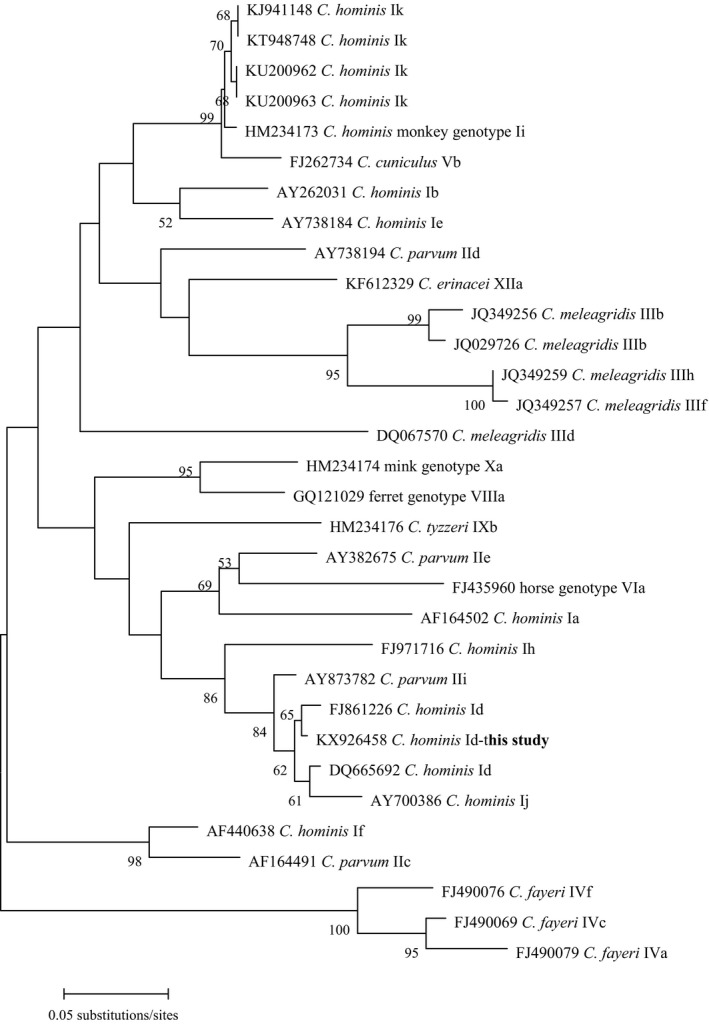
Phylogenetic relationship of 60‐kDa glycoprotein (*GP60*) nucleotide sequences of the *Cryptosporidium* equine isolate in this study to multiple subtype families in *C. hominis*, as inferred by a neighbor‐joining analysis based on evolutionary distances calculated using the Kimura two‐parameter model. The tree was rooted with partial subtypes of *C. fayeri*. Bootstrap values were obtained using 1,000 pseudo‐replicates, with only values above 50% reported.

In a previous study, *C. hominis* was first identified in only one horse in Algeria, and a novel *GP60* subtype family, Ik, was identified (Laatamna et al. 2015). In Brazil, *C. hominis* was reported in two foals of 4 and 5 mo of age, respectively, and one subtype IkA20G1 was found (Inácio et al. 2017). In contrast, *C. hominis* was identified as the dominant parasite (61/87, 70.1%) in horses in China, and two subtypes were found: IkA16G1 and IkA16 (Jian et al. 2016). In the present study, two isolates of *C. hominis* were successfully amplified at the *GP60* locus and were identified for subtypes. DNA sequence analysis showed that the two isolates have a maximum nucleotide identity of 99% to a previously characterized Australian *C. hominis* subtype, IdA26 (FJ861226). These subtypes have less than 11 nucleotide TCA repeats and five nucleotide substitutions: A/G, G/A, T/C, C/A, and T/C at nucleotides 182, 258, 316, 484, and 835, respectively. The two isolates were determined to belong to the Id subtype based on phylogenetic analysis, and were named IdA15 in accordance with current *Cryptosporidium* nomenclature conventions (Fig. 1) (Sulaiman et al. 2005). Human infections with subtype Id *C. hominis* have previously been reported in China, Canada (Ontario and British Columbia), and the Arctic, and no animal‐derived subtype Id isolates have been described to date (Karine et al. 2016; Ong et al. 2008; Trotzwilliams et al. 2006; Wang et al. 2011). The subtype Id of *C. hominis* has been detected in humans in the area of Tianjin and Henan province in China (Wang et al. 2011). The two isolates found in horses were from Farm 2 in this area. On this farm close contact between people and horses may be responsible for infection of the horses with *C. hominis*. Whether these horses are truly infected or merely act as mechanical carriers of accidentally ingested oocysts of anthroponotic origin remains to be determined.

*Cryptosporidium andersoni* was considered the major species causing bovine cryptosporidiosis in previous studies (Liu et al. 2015; Zhao et al. 2015), but it has been identified in various hosts in China, including humans, golden takins, bactrian camels, cattle, sheep, goats, and yaks (Liu et al. 2014a,b; Qi et al. 2015; Zhao et al. 2015). Each *C. andersoni*‐positive isolate was successfully amplified and sequenced at the four minisatellite loci: MS1, MS2, MS3, and MS16. Multiple‐sequence alignment analysis of the four *C. andersoni*‐positive sequences showed A6, A5, A2, and A1 at the four loci, respectively. This MLST subtype was first reported in Bactrian camels in the Henan province, and has also been reported in Bactrian camels in the Sichuan province in China (Wang et al. 2012).

In summary, this is the first report of *C. hominis* and *C. andersoni* in horses in southwestern China, as well as the first report of the rare zoonotic subtype Id of *C. hominis* and the MLST subtypes A6, A5, A2, and A1 of *C. andersoni* in horses overall. *Cryptosporidium andersoni* has been identified in other animals, and the MLST subtypes A6, A5, A2, and A1 have also been reported in other animals in southwestern China. It is therefore likely that the horse‐derived *C. andersoni* resulted from cross‐species transmission. Importantly, the presence of the *C. hominis* Id subtype in both humans and horses indicates that it may present a threat to public health.

## Supporting information


**Table S1.** Gene locus and primer sequences used in this study, annealing temperatures used in the PCR and expected sizes of the PCR products.

## References

[jeu12399-bib-0001] Burton, A. J. , Nydam, D. V. , Dearen, T. K. , Mitchell, K. , Bowman, D. D. & Xiao, L. 2010 The prevalence of *Cryptosporidium*, and identification of the *Cryptosporidium* horse genotype in foals in New York State. Vet. Parasitol., 174:139–144. 20932647 10.1016/j.vetpar.2010.08.019

[jeu12399-bib-0002] Cacciò, S. M. 2005 Molecular epidemiology of human cryptosporidiosis. Parassitologia, 47:185–192. 16252472

[jeu12399-bib-0003] Current, W. L. , Reese, N. C. , Ernst, J. V. , Bailey, W. S. , Heyman, M. B. & Weinstein, W. M. 1983 Human cryptosporidiosis in immunocompetent and immunodeficient persons. Studies of an outbreak and experimental transmission. N. Engl. J. Med., 308:1252–1257. 6843609 10.1056/NEJM198305263082102

[jeu12399-bib-0004] Fayer, R. , Morgan, U. & Upton, S. J. 2000 Epidemiology of *Cryptosporidium*: transmission, detection and identification. Int. J. Parasitol., 30:1305–1322. 11113257 10.1016/s0020-7519(00)00135-1

[jeu12399-bib-0005] Feng, Y. , Yang, W. , Ryan, U. , Zhang, L. , Kváč, M. , Koudela, B. , Modrý, D. , Li, N. , Fayer, R. & Xiao, L. 2011 Development of a multilocus sequence tool for typing *Cryptosporidium muris* and *Cryptosporidium andersoni* . J. Clin. Microbiol., 49:34–41. 20980577 10.1128/JCM.01329-10 PMC3020410

[jeu12399-bib-0006] Graaf, D. C. D. , Vanopdenbosch, E. , Ortega‐Mora, L. M. , Abbassi, H. & Peeters, J. E. 1999 A review of the importance of cryptosporidiosis in farm animals. Int. J. Parasitol., 29:1269–1287. 10576578 10.1016/S0020-7519(99)00076-4 PMC7127282

[jeu12399-bib-0007] Inácio, S. V. , Widmer, G. , de Brito, R. L. , Zucatto, A. S. , de Aquino, M. C. , Oliveira, B. C. , Nakamura, A. A. , Neto, L. D. , Carvalho, J. G. , Gomes, J. F. , Meireles, M. V. & Bresciani, K. D. 2017 First description of *Cryptosporidium hominis GP60* genotype IkA20G1 and *Cryptosporidium parvum GP60* genotypes IIaA18G3R1 and IIaA15G2R1 in foals in Brazil. Vet. Parasitol., 233:48–51. 28043388 10.1016/j.vetpar.2016.11.021

[jeu12399-bib-0008] Jian, F. , Liu, A. , Wang, R. , Zhang, S. , Qi, M. , Zhao, W. , Shi, Y. , Wang, J. , Wei, J. & Zhang, L. 2016 Common occurrence of *Cryptosporidium hominis* in horses and donkeys. Infect. Genet. Evol., 43:261–266. 27264727 10.1016/j.meegid.2016.06.004

[jeu12399-bib-0009] Karim, M. R. , Wang, R. , He, X. , Zhang, L. , Li, J. , Rume, F. I. , Dong, H. , Qi, M. , Jian, F. , Zhang, S. , Sun, M. , Yang, G. , Zou, F. , Ning, C. & Xiao, L. 2014 Multilocus sequence typing of *Enterocytozoon bieneusi* in nonhuman primates in China. Vet. Parasitol., 200:13–23. 24388499 10.1016/j.vetpar.2013.12.004

[jeu12399-bib-0010] Karine, T. , Asma, I. , Brent, D. , Réjean, D. , Benoît, L. , Philippe, C. , Lyne, C. , Momar, N. , Jean‐François, P. & Yansouni, C. P. 2016 *Cryptosporidium hominis* is a newly recognized pathogen in the Arctic region of Nunavik, Canada: molecular characterization of an outbreak. PLoS Negl. Trop Dis., 10(4):e0004534. 27058742 10.1371/journal.pntd.0004534 PMC4825996

[jeu12399-bib-0011] Kenzie, W. R. M. , Schell, W. L. , Blair, K. A. , Addiss, D. G. , Dan, E. P. , Hoxie, N. J. , Kazmierczak, J. J. & Davis, J. P. 1995 Massive outbreak of waterborne *Cryptosporidium* infection in Milwaukee, Wisconsin: recurrence of illness and risk of secondary transmission. Clin. Infect. Dis., 21:57–62. 7578760 10.1093/clinids/21.1.57

[jeu12399-bib-0012] Laatamna, A. E. , Wagnerová, P. , Sak, B. , Květoňová, D. , Xiao, L. , Rost, M. , Mcevoy, J. , Saadi, A. R. , Aissi, M. & Kváč, M. 2015 Microsporidia and *Cryptosporidium* in horses and donkeys in Algeria: detection of a novel *Cryptosporidium hominis* subtype family (Ik) in a horse. Vet. Parasitol., 208:135–142. 25638716 10.1016/j.vetpar.2015.01.007

[jeu12399-bib-0013] Liu, A. , Jia, Z. , Zhao, J. , Wei, Z. , Wang, R. & Zhang, L. 2015 The first report of *Cryptosporidium andersoni* in horses with diarrhea and multilocus subtype analysis. Parasit. Vectors, 8:1–4. 26394848 10.1186/s13071-015-1102-0 PMC4580357

[jeu12399-bib-0014] Liu, H. , Shen, Y. , Yin, J. , Yuan, Z. , Jiang, Y. , Xu, Y. , Pan, W. , Hu, Y. & Cao, J. 2014a Prevalence and genetic characterization of *Cryptosporidium*,* Enterocytozoon, Giardia* and *Cyclospora* in diarrheal outpatients in China. BMC Infect. Dis., 14:290–292. 24410985 10.1186/1471-2334-14-25 PMC3925443

[jeu12399-bib-0015] Liu, X. , Zhou, X. , Zhong, Z. , Deng, J. , Chen, W. , Cao, S. , Fu, H. , Zuo, Z. , Hu, Y. & Peng, G. 2014b Multilocus genotype and subtype analysis of *Cryptosporidium andersoni* derived from a Bactrian camel (*Camelus bactrianus*) in China. Parasitol. Res., 113:2129–2136. 24676462 10.1007/s00436-014-3863-3

[jeu12399-bib-0016] Ong, C. S. , Chow, S. , Gustafson, R. , Plohman, C. , Parker, R. , Isaac‐Renton, J. L. & Fyfe, M. W. 2008 Rare *Cryptosporidium hominis* subtype associated with aquatic center use. Emerg. Infect. Dis., 14:1323–1325. 18680673 10.3201/eid1408.080115 PMC2600396

[jeu12399-bib-0017] Panciera, R. J. , Thomassen, R. W. & Garner, F. M. 1970 Cryptosporidial infection in a calf. Vet. Pathol., 8:479–484.

[jeu12399-bib-0018] Petri, W. A. , Miller, M. , Binder, H. J. , Levine, M. M. , Dillingham, R. & Guerrant, R. L. 2008 Enteric infections, diarrhea, and their impact on function and development. J. Clin. Invest., 118:1277–1290. 18382740 10.1172/JCI34005 PMC2276781

[jeu12399-bib-0019] Qi, M. , Wang, H. , Jing, B. , Wang, D. , Wang, R. & Zhang, L. 2015 Occurrence and molecular identification of *Cryptosporidium* spp. in dairy calves in Xinjiang, Northwestern China. Vet. Parasitol., 212:404–407. 26186985 10.1016/j.vetpar.2015.07.002

[jeu12399-bib-0020] Ryan, U. , Fayer, R. & Xiao, L. 2014 *Cryptosporidium* species in humans and animals: current understanding and research needs. Parasitology, 141:1–19. 25111501 10.1017/S0031182014001085

[jeu12399-bib-0021] Ryan, U. , Paparini, A. , Tong, K. , Yang, R. , Gibson‐Kueh, S. , O'Hara, A. , Lymbery, A. & Xiao, L. 2015 *Cryptosporidium* huwi n. sp. (*Apicomplexa: Eimeriidae*) from the guppy (*Poecilia reticulata*). Exp. Parasitol., 150:31–35. 25637783 10.1016/j.exppara.2015.01.009

[jeu12399-bib-0022] Slavin, D. 1955 *Cryptosporidium* meleagridis (sp. nov.). J. Comp. Pathol. Ther., 65:262–266. 10.1016/s0368-1742(55)80025-2 13242675

[jeu12399-bib-0023] Snyder, S. P. , England, J. J. & Mcchesney, A. E. 1978 Cryptosporidiosis in immunodeficient Arabian foals. Vet. Pathol., 15:12–17. 625861 10.1177/030098587801500102

[jeu12399-bib-0024] Sulaiman, I. M. , Hira, P. R. , Zhou, L. , Alali, F. M. , Alshelahi, F. A. , Shweiki, H. M. , Iqbal, J. , Khalid, N. & Xiao, L. 2005 Unique endemicity of cryptosporidiosis in children in Kuwait. J. Clin. Microbiol., 43:2805–2809. 15956401 10.1128/JCM.43.6.2805-2809.2005 PMC1151898

[jeu12399-bib-0025] Sulaiman, I. M. , Morgan, U. M. , Thompson, R. C. A. , Lal, A. A. & Xiao, L. 2000 Phylogenetic relationships of *Cryptosporidium* parasites based on the 70‐kilodalton heat shock protein (*HSP70*) gene. Appl. Environ. Microbiol., 66:2385–2391. 10831415 10.1128/aem.66.6.2385-2391.2000 PMC110539

[jeu12399-bib-0026] Trotzwilliams, L. A. , Martin, D. S. , Gatei, W. , Cama, V. , Peregrine, A. S. , Martin, S. W. , Nydam, D. V. , Jamieson, F. & Xiao, L. 2006 Genotype and subtype analyses of *Cryptosporidium* isolates from dairy calves and humans in Ontario. Parasitol. Res., 99:346–352. 16565813 10.1007/s00436-006-0157-4

[jeu12399-bib-0027] Tyzzer, E. E. 1907 A sporozoan fund in the peptic glands of the common mouse. Proc. Soc. Exp. Biol. Med., 56:12–13.

[jeu12399-bib-0028] Wagnerová, P. , Sak, B. , Mcevoy, J. , Rost, M. , Matysiak, A. P. , Ježková, J. & Kváč, M. 2015 Genetic diversity of *Cryptosporidium* spp. including novel identification of the *Cryptosporidium muris* and *Cryptosporidium tyzzeri* in horses in the Czech Republic and Poland. Parasitol. Res., 114:1619–1624. 25722018 10.1007/s00436-015-4353-y

[jeu12399-bib-0029] Wang, R. , Jian, F. , Zhang, L. , Ning, C. , Liu, A. , Zhao, J. , Feng, Y. , Qi, M. , Wang, H. & Lv, C. 2012 Multilocus sequence subtyping and genetic structure of *Cryptosporidium muris* and *Cryptosporidium andersoni* . PLoS ONE, 7:e43782. 22937094 10.1371/journal.pone.0043782 PMC3427161

[jeu12399-bib-0030] Wang, R. , Zhang, X. , Zhu, H. , Zhang, L. , Feng, Y. , Jian, F. , Ning, C. , Qi, M. , Zhou, Y. & Fu, K. 2011 Genetic characterizations of *Cryptosporidium* spp. and *Giardia duodenalis* in humans in Henan, China. Exp. Parasitol., 127:42–45. 20599984 10.1016/j.exppara.2010.06.034

[jeu12399-bib-0031] Xiao, L. , Bern, C. , Limor, J. , Sulaiman, I. , Roberts, J. , Checkley, W. , Cabrera, L. , Gilman, R. H. & Lal, A. A. 2001 Identification of 5 types of *Cryptosporidium* parasites in children in Lima, Peru. J. Infect. Dis., 183:492–497. 11133382 10.1086/318090

[jeu12399-bib-0032] Xiao, L. , Escalante, L. , Yang, C. , Sulaiman, I. , Escalante, A. A. , Montali, R. J. , Fayer, R. & Lal, A. A. 1999 Phylogenetic analysis of *Cryptosporidium* parasites based on the small‐subunit rRNA gene locus. Appl. Environ. Microbiol., 65:1578–1583. 10103253 10.1128/aem.65.4.1578-1583.1999 PMC91223

[jeu12399-bib-0033] Xiao, L. H. , Limor, J. , Morgan, U. M. , Sulaiman, I. M. , Thompson, R. C. A. & Lal, A. A. 2000 Sequence differences in the diagnostic target region of the oocyst wall protein gene of *Cryptosporidium* parasites. Appl. Environ. Microbiol., 66:5499–5502. 11097936 10.1128/aem.66.12.5499-5502.2000 PMC92490

[jeu12399-bib-0034] Zhao, G. H. , Du, S. Z. , Wang, H. B. , Hu, X. F. , Deng, M. J. , Yu, S. K. , Zhang, L. X. & Zhu, X. Q. 2015 First report of zoonotic *Cryptosporidium spp*., *Giardia intestinalis* and *Enterocytozoon bieneusi* in golden takins (*Budorcas taxicolor bedfordi*). Infect. Genet. Evol., 34:394–401. 26190449 10.1016/j.meegid.2015.07.016

