# Supplementary material for: Occurrence and Genetic Characteristics of Cryptosporidium hominis and Cryptosporidium andersoni in Horses from Southwestern China
Source: J Eukaryot Microbiol. 2017 Mar 8;64(5):716–20. doi: 10.1111/jeu.12399 (PMC5599969; doi:10.1111/jeu.12399)
Supplement: Supplementary file 1 — Table S1. Gene locus and primer sequences used in this study, annealing temperatures used in the PCR and expected sizes of the PCR products. [file JEU-64-716-s001.pdf]

SUPPORTING INFORMATION

Occurrence and Genetic Characteristics of *Cryptosporidium hominis* and *Cryptosporidium andersoni* in Horses from Southwestern China by Lei Deng, Wei Li, Zhijun Zhong, Chao Gong, Xuefeng Cao, Yuan Song, Wuyou Wang, Xiangming Huang, Xuehan Liu, Yanchun Hu, Hualin Fu, Min He, Ya Wang, Yue Zhang, Kongju Wu & Guangneng Peng

Table S1. Gene locus and primer sequences used in this study, annealing temperatures used in the PCR and expected sizes of the PCR products

Table S1. Gene locus and primer sequences used in this study, annealing temperatures used in the PCR and expected sizes of the PCR products

Gene locus	Primer sequences (5′-3′)	Annealing temperature (°C)	Fragment length (bp)	References
SSU rRNA	F1:TTCTAGAGCTAATACATGCG	55	1325	Xiao et al. 1999
	R1:CCCATTTCCTTCGAAACAGGA			
	F2:GGAAGGGTTGTATTTATTAGATAAAG	58	820	
	R2: AAGGAGTAAGGAACAACCTCCA			
HSP70	F1:ATGTCTGAAGGTCCAGCTATTGGTATTGA	55	2015	Sulaiman et al. 2000
	R1: TTAGTCGACCTCTTCAACAGTTGG			
	F2:TATTCATGTGTTGGTGTATGGAGAAA	45	1950	
	R2:CAACAGTTGGACCATTAGATCC			
COWP	F1:CCCAACATTCCTGGTGTAGCTTCC	56	1033	Xiao et al. 2000
	R1:CCCAACATTCCTGGTGTAGCTTCC			
	F2:GTAGATAATGGAAGAGATTGTG	56	553	
	R2:GGACTGAAATACAGGCATTATCTTG			
GP60	F1:ATAGTCTCCGCTGTATTC	55	870	Sulaiman et al. 2005
	R1:TCCGCTGTATTCTCAGCC			
	F2:GGAAGGAACGATGTATCT	58	850	
	R2:GCAGAGGAACCAGCATC			
MS1	F1:ACCATCTAGAGATAACGAGCGA	55	660	Feng et al. 2011
	R1:GAATCAGAAGATGAGCGACAA			
	F2:CGTGATAGTGGGTATGAATTGGACA	55	550	
	R2:CGACTGCGATACTCACGTCCT			
MS2	F1:TTGCAACTGTACCTAAATTAGTA	55	500	Feng et al. 2011
	R1:GTGAGACTTCTGGGGTCCTGA			
	F2:TCATGACGCGTCATACCAACA	52	457	
	R2:ACTTAGACAGTTCTATGCTGA			
MS3	F1:AACCAAGTGAATCACGAACTT	55	574	Feng et al. 2011
	R1:TCAAGTACAGCAGTCTATTGCTT			
	F2:GCAATATCTTCGACGATCCCA	55	536	
	R2:ATGGGAATAATTCTTCATCATCAA			
MS16	F1:GAAGAGGTCGAAGTTAAGCTA	55	620	Feng et al. 2011
	R1:GACAATCATCTAAATCGTGTT			
	F2:AAGTTTCATCTAGGTACACTAAGA	55	597	
	R2:CACTACCTAATCTCGTGTACTT			
